# Europium (III) chelate microparticle-based lateral flow immunoassay strips for rapid and quantitative detection of antibody to hepatitis B core antigen

**DOI:** 10.1038/s41598-017-14427-4

**Published:** 2017-10-26

**Authors:** Rong-Liang Liang, Qiao-Ting Deng, Zhen-Hua Chen, Xu-Ping Xu, Jian-Wei Zhou, Jun-Yu Liang, Zhi-Ning Dong, Tian-Cai Liu, Ying-Song Wu

**Affiliations:** 1 Institute of Antibody Engineering, School of Laboratory Medicine and Biotechnology, Southern Medical University, Guangzhou, 510515 P.R. China; 2 Guangdong Provincial Key Laboratory of Tropical Disease Research, School of Public Health, Southern Medical University, Guangzhou, 510515 P.R. China

## Abstract

Quantitative hepatitis B core antigen (anti-HBc) measurements could play an important role in evaluating therapeutic outcomes and optimizing the antiviral therapy of chronic hepatitis B infection. In this study, we have developed a simple and rapid fluorescence point-of-care test based on a lateral flow immunoassay (LFIA) method integrated with Eu (III) chelate microparticles to quantitatively determine anti-HBc concentrations in serum. This assay is based on a direct competitive immunoassay performed on lateral flow test strips with an assay time of 15 min. The Eu (III) chelate microparticle-based LFIA assay could quantitatively detect anti-HBc levels with a limit of detection of 0.31 IU mL^−1^, and exhibited a wide linear range (0.63–640 IU mL^−1^). The intra- and inter-assay coefficients of variation for anti-HBc were both less than 10% and a satisfactory dilution test and accuracy were demonstrated. There were no statistically significant differences in sensitivity or specificity in serum samples between the Eu (III) chelate microparticle-based LFIA strips and the Abbott Architect kit. A simple, rapid and effective quantitative detection of anti-HBc was possible using the Eu (III) chelate microparticle-based LFIA strips. The strips will provide diagnostic value for clinical application.

## Introduction

Hepatitis B virus (HBV) infection is a global public health problem, which has a wide range of clinical consequences, from acute and chronic infection to severe chronic liver disease, including cirrhosis and hepatocellular carcinoma^1–3^. Antibody to hepatitis B core antigen (anti-HBc) is one of the earliest serological markers during HBV infection, appearing during the acute phase of HBV infection and persisting thereafter^4^. Generally, the presence of anti-HBc is considered to be an important indicator of both existing and past HBV infection, and is routinely used in clinical diagnosis or blood screening in countries where hepatitis B is endemic, such as China^5,6^. However, the clinical significance of quantitative measurements of anti-HBc levels is still largely unknown.

Recently, with some immunoassays being validated using the World Health Organization anti-HBc standards, the clinical significance of quantitative anti-HBc (qAnti-HBc) measurements has been increasingly investigated^7^. Serum qAnti-HBc levels have been shown to vary during different phases of HBV infection, which is determined by host immune status, and they are strongly associated with hepatic inflammation in persons with chronic HBV infection^8,9^. In addition, qAnti-HBc could be useful as a marker of HBV-induced liver-disease to discriminate between major phases of chronic HBV infection and to predict a sustained response to antiviral therapies^10^. Moreover, baseline anti-HBc levels, combining vascular invasion and alpha-fetoprotein (AFP) levels might be a novel biomarker for predicting the survival of hepatocellular carcinoma patients after transarterial chemoembolization^11^. Furthermore, baseline anti-HBc levels can predict the hepatitis B e antigen (HBeAg) seroconversion rate, which can be used as a reliable predictor of the efficacy of peginterferon and nucleos(t)ide analogues therapy, and could be used for optimizing the antiviral therapy of chronic hepatitis B infection (CHB)^12–14^. Therefore, qAnti-HBc measurements could play an important role in diagnosing, evaluating therapeutic outcomes and optimizing the antiviral therapy of CHB. In addition, they will help to create a deeper understanding of the natural course of CHB.

To date, most commercially available anti-HBc kits and reported detection methods are qualitative assays used in clinical practice, only a few kits are quantitative assays, limiting the scope for quantitative applications^15–18^. Existing studies of the clinical significance of qAnti-HBc were performed using a double-antigen sandwich enzyme-linked immunosorbent assay (ELISA), which was validated using the international anti-HBc standards^7^. Although the ELISA produced a quantitative result with high sensitivity and excellent specificity, there are still some inconveniences and shortcomings such as the complexity of the procedures, relative expensive and long operation times. Therefore, a simple, rapid and quantitative assay for anti-HBc is desirable. In recent years, the lateral flow immunoassay (LFIA), a well-established and accepted point-of-care testing (POCT) technique, has been used to develop quantitative fluorescent strips using fluorescent reporters including colloidal gold, fluorescent dyes, quantum dots and lanthanide chelates^19–22^. In our previous studies, we reported that LFIA based Polystyrene Eu (III) chelate microparticles could be used for quantitative detection of AFP and creatine kinase MB^23,24^. The LFIA system not only takes advantage of the simplicity, rapidity and relatively low cost of conventional lateral flow strips, but also possesses a high sensitivity afforded by the polystyrene Eu (III) chelate microparticles owing to their characteristic narrow band emission and large Stokes shift^25^.

In the current study, like most commercially available anti-HBc kits that adopt a competitive format, we used carboxylate-modified polystyrene Eu (III) chelate microparticles (CM-EUs) as a reporter to establish a direct competitive LFIA system for the detection of qAnti-HBc in human serum. The ratio of the fluorescence peak heights of test line and control line (H_T_/H_C_ ratio) was applied to quantify the result of the measurements. The performance of the polystyrene Eu (III) chelate microparticle-based LFIA, including its linearity, analytical sensitivity, reproducibility, accuracy and cross-reactivity, were evaluated. In addition, we employed the polystyrene Eu (III) chelate microparticle-based LFIA to test 343 clinical samples. The experimental results show that the proposed method is highly suited for the quantitative analysis of anti-HBc levels.

## Results

### Principle of the method

**Figure 1:**
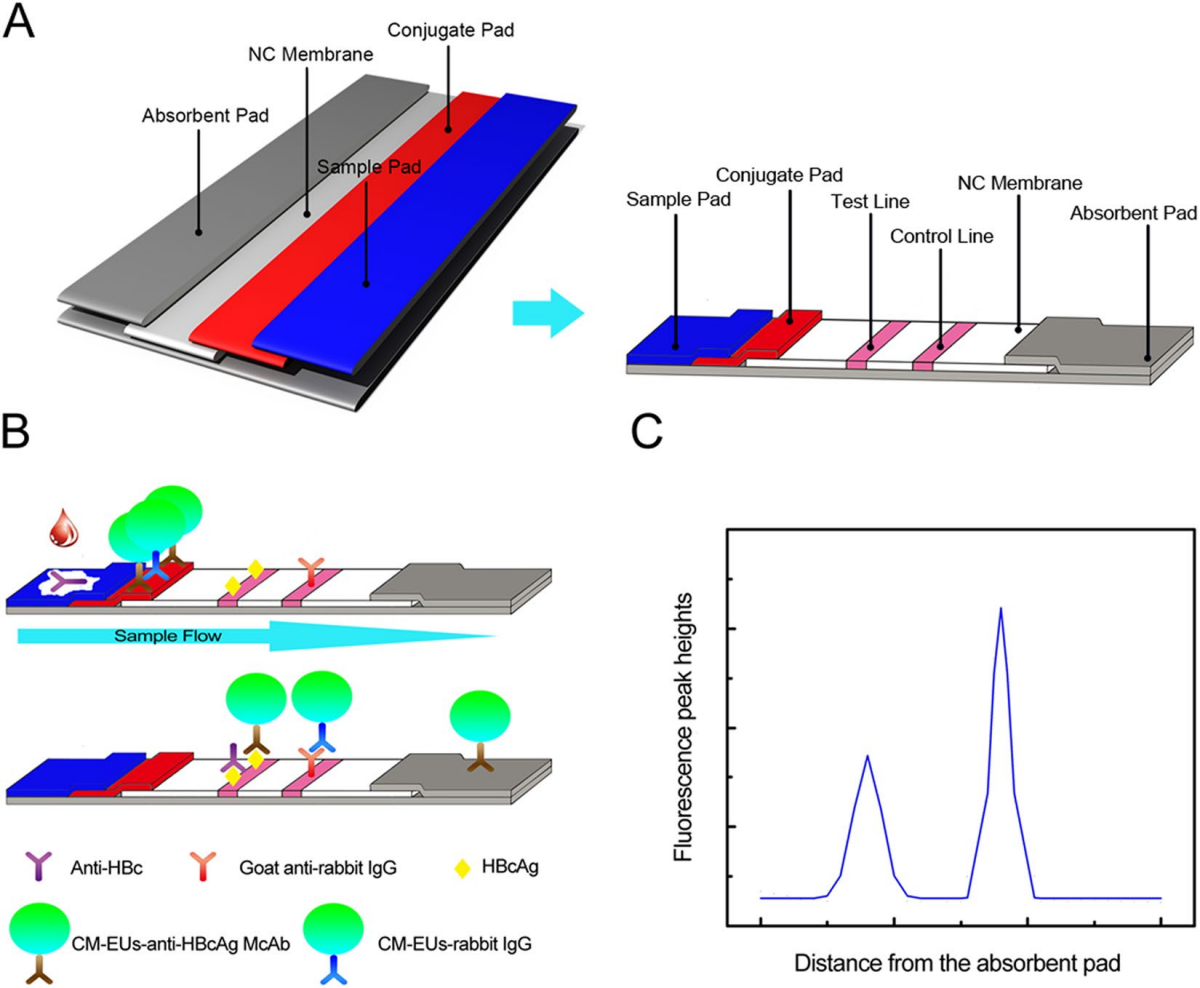
Schematic illustration of the CM-EUs-based lateral flow assay. (**A**) Components and assembly of CM-EUs-based lateral flow test strip. (**B**) The recombinant HBcAg and the anti-RIgG were immobilized on the test line and the control line, respectively. Samples containing anti-HBc are added to the sample pads and migrate along the NC membrane by capillary action, and the anti-HBcAg McAb competes with the anti-HBc in the sample for binding to the immobilized protein. (**C**) The fluorescence peak height is detected using a portable TRF strip reader.

The proposed lateral flow assay for determining anti-HBc levels was performed as described for a typical competitive time-resolved fluoroimmunoassay, as illustrated in Fig. 1B. With the aid of the capillarity of the absorbent pad, the sample buffer containing anti-HBc was added onto the sample pad and the conjugates of CM-EUs with anti-HBcAg McAb migrated across the NC membrane and reacted with the HBcAg on the test line while the conjugates of CM-EUs with RIgG were captured by anti-RIgG coated on the control line, resulting in a fluorescent band on the test and control lines, respectively. Subsequently, the excess fluorescent microspheres migrated into the absorption pad. After completion of the reaction, the test strip was assessed using a TRF reader by measuring the peak heights of the test line and the control line (Fig. 1C). In the competition assay system, the conjugates of CM-EUs with anti-HBcAg McAb compete with anti-HBc in the sample for binding to the coated recombinant HBcAg, meaning that the more anti-HBc in the sample, the lower the fluorescence intensity appears on the test line. Therefore, the fluorescence intensity at the test line is inversely related to the concentration of anti-HBc in the sample. In contrast, as the internal control, the fluorescence intensity at the control line was almost constant, regardless of anti-HBc concentrations in the sample, which confirmed that the sample migrated through the lines and that the assay functioned correctly. Lastly, the H_T_/H_C_ ratio was used for the measurements, which could counteract the effects of the sample matrix and the inherent heterogeneity of the test strips, increasing the accuracy of the method and rendering it more credible for clinical application^26,27^.

### Dilution test

**Figure 2:**
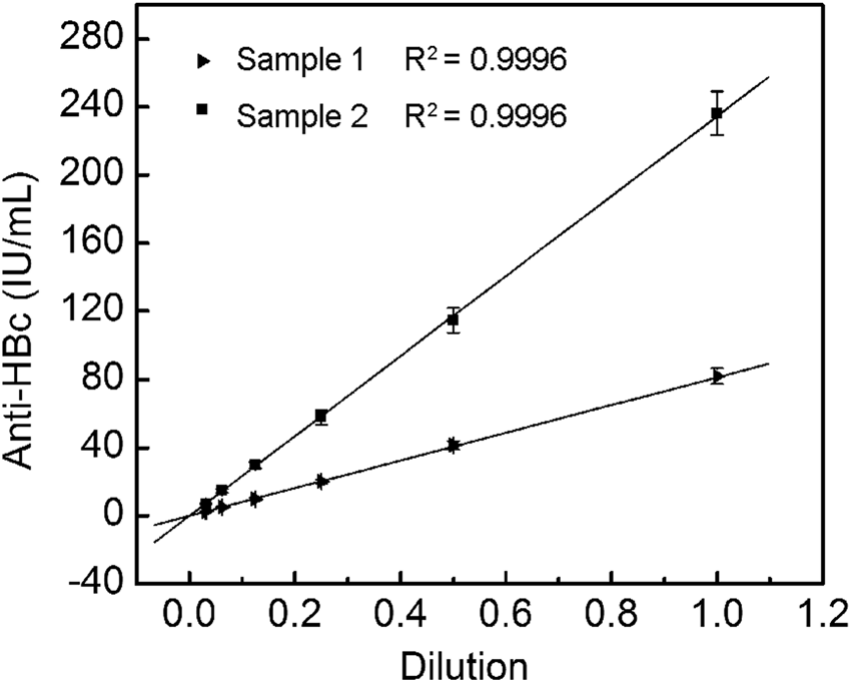
Dilution linearity for anti-HBc based on the measurement of two anti-HBc positive serum samples.

A dilution test was conducted to confirm the reliability of the assay using a serial dilution from 1/2 to 1/32 with standard buffer for two positive sera samples. As shown in Fig. 2, both sample dilutions demonstrated good linearity (both R square values are above 0.999), manifesting little variation in the observed concentrations after correcting for sample dilution elements. This demonstrated that the proposed method was feasible for quantitative measurements.

### Linearity and analytical sensitivity

**Figure 3:**
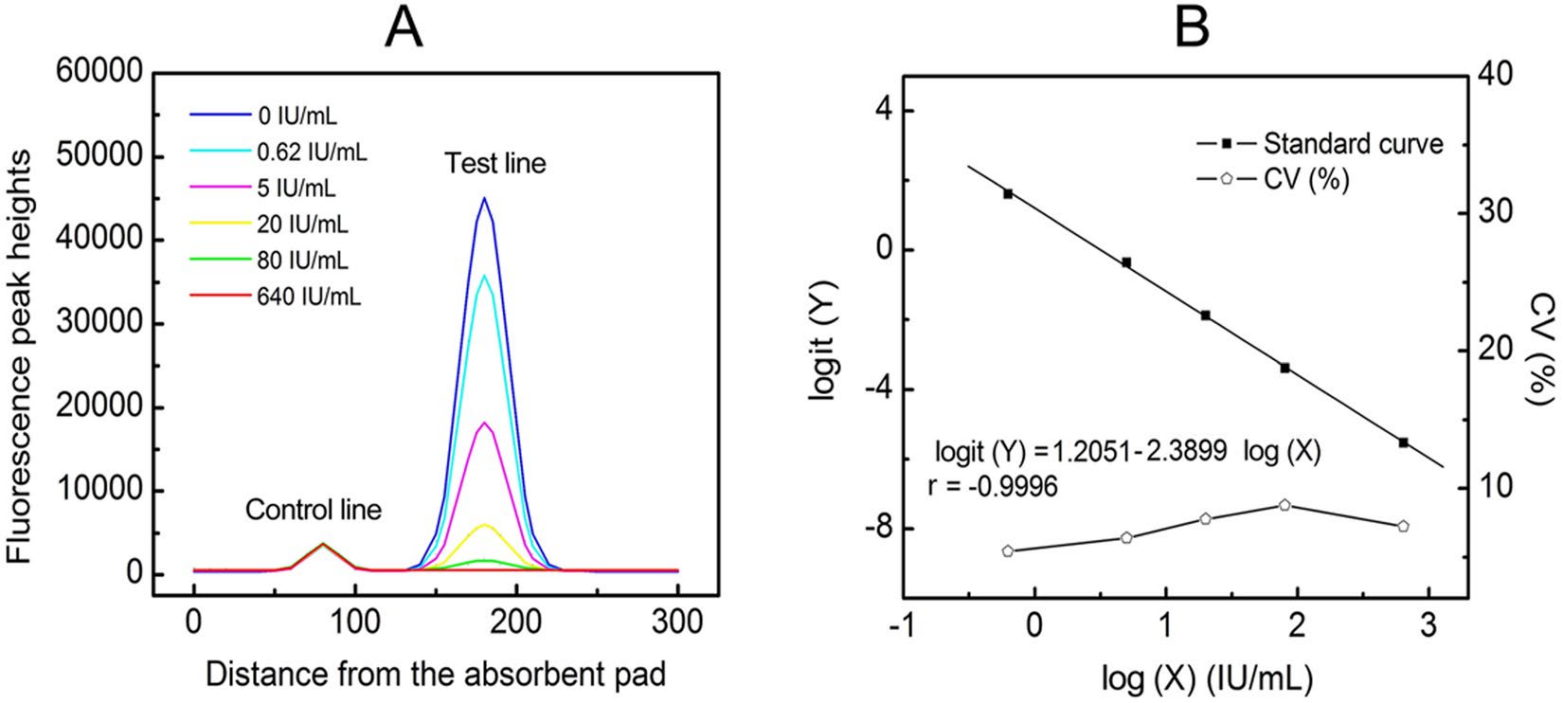
Fluorescence peak heights readout curve and Standard curve of CM-EUs-based LFIA strips for anti-HBc. (**A**) Fluorescence peak heights readout curve for anti-HBc at concentration of 0 to 640 IU mL^−1^. (**B**) Standard curve of CM-EUs-based LFIA strips for anti-HBc was obtained for calibration samples from 0.63 to 640 IU mL^−1^ and the intra-assay CV% for each data point based on five replicates. A logit-log plot was obtained from the computation formula: logit (Y) = ln [(B_x_/B_0_)/(1 − B_x_/B_0_)].

The standard curve for the assay was constructed based on the measurement of a series of different concentrations of anti-HBc standards (0, 0.63, 5, 20, 80 and 640 IU mL^−1^), which were accurately prepared in sample buffer. The fluorescence peak heights readout curves of the TRF strip reader are shown in Fig. 3A. After recording the fluorescence intensities, we obtained a standard curve by plotting the logit (Y) against the logarithm of the anti-HBc concentration as represented by the equation: logit (Y) = 1.2051–2.3899 log (X), with a reliable correlation coefficient (r = −0.9996) and linearity was displayed throughout the entire anti-HBc range as shown in Fig. 3B. At each concentration, the coefficients of variation (CVs) were less than 10% based on five duplicated measurements (Fig. 3B). The analytical sensitivity (limit of detection), defined as the concentration that corresponds to the mean minus 2 × SD (n = 20) of the H_T_/H_C_ ratio of the zero standard, was calculated to be 0.31 IU mL^−1^.

### Precision

The intra-assay (within day) and inter-assay (between days) precision was evaluated to determine the reproducibility of the developed immunoassay. Three concentrations of anti-HBc in sera samples (weakly positive, positive, strongly positive) were measured 10 times per day to determine intra-assay precision, and measurements were performed on 3 sequential days with 10 replicates for the inter-assay precision. The results are shown in Table 1. The intra- and inter-assay CVs are 5.63% to 7.79% (n = 10) and 6.93% to 9.82% (n = 30), respectively. All the CVs are below 10%, which demonstrates an acceptable level of precision for the anti-HBc strip quantification.

**Table 1:** The intra- and inter-assay precision.

Samples	Intra-assay precision (n = 10)		Inter-assay precision (n = 30)	
	Mean ± SD (IU mL^−1^)	CV%	Mean ± SD (IU mL^−1^)	CV%
1	4.74 ± 0.39	7.79	4.69 ± 0.46	9.82
2	24.75 ± 1.79	6.74	26.02 ± 2.21	8.48
3	176.76 ± 9.95	5.63	185.62 ± 12.86	6.93

### Accuracy

To evaluate the accuracy of the proposed method, three samples consisting of different concentrations of anti-HBc were prepared using certified anti-HBc standard materials from the NIBSC in standard buffer at concentrations of 1.56 IU mL^−1^, 12.5 IU mL^−1^ and 50 IU mL^−1^. The three samples were measured five times using the polystyrene Eu (III) chelate microparticle-based LFIA system and the relative deviations (RDs) of the analytes were calculated using the equation: RD = (measured values − theoretical value)/theoretical value. As shown in Table 2, the RDs of anti-HBc ranged from −7.06% to −2.88%, with a CV ranging from 6.21% to 7.33%. These results demonstrate that the accuracy of the assay is acceptable for clinical analytical purposes.

**Table 2:** Relative deviations of our novel assay for three concentrations of NIBSC anti-HBc certified standard materials.

Sample (IU mL^−1^)	Mean ± SD (IU mL^−1^)	CV % (n = 5)	Relative deviation %
1.56	1.45 ± 0.09	6.21	−7.06
12.25	12.14 ± 0.89	7.33	−2.88
50	47.88 ± 3.20	6.69	−4.24

### Cross-reactivity and interference experiments

Cross-reactivity studies were performed using 94 specimens positive for anti-HAV, anti-HCV, anti-HEV anti-HIV, anti-HSV, anti-CMV, anti-EBV, anti-HTLV, anti-nuclear antibody, systemic lupus erythematosus, rheumatoid factor, toxoplasma gondii IgM, syphilis, rubella antibody, *E. coli* infection and yeast infection. Using these specimens, the anti-HBc LFIA and commercially available diagnostic kits (Abbott CMIA) showed 100% agreement (94/94). Moreover, the LFIA assay was unaffected by triglycerides (≤200 mg dL^−1^), bilirubin (≤8 mg dL^−1^), and hemoglobin (≤400 mg dL^−1^). These results demonstrate that the assay developed in the current study has a high specificity, and is not influenced by these common interferents.

### Anti-HBc measurements in serum samples

To define the reference range of anti-HBc levels using this LFIA method, we analyzed serum samples from 112 healthy people using the Eu (III) chelate microparticle-based LFIA strips (see Supplementary Table S1). A reference range of 0 to 1.0 IU mL^−1^ anti-HBc (95% confidence interval) was possible, meaning that samples were considered negative if the concentration was <1.0 IU mL^−1^ and positive if the concentration was ≥1.0 IU mL^−1^.

In order to demonstrate the clinical application of the polystyrene Eu (III) chelate microparticle-based LFIA system, we tested a further 231 serum specimens (see Supplementary Data Table S2). We compared the data with those obtained from the Architect anti-HBc II reagent kit (Abbott Laboratories; Table 3) and analyzed the sensitivity and specificity of the assays using McNemar’s test with SPSS13.0 statistical software. In the Eu (III) chelate microparticle-based LFIA, the sensitivity and specificity values for anti-HBc detection were 95.90% (117/122) and 99.08% (108/109), respectively, with respective kappa values of 0.948 (P < 0.001). There were no statistically significant differences in sensitivity or specificity between the Eu (III) chelate microparticle-based LFIA strips and the Abbott Architect kits, and the LFIA strips displayed a good consistency.

**Table 3:** McNemar’s test between the proposed method and CMIA for the test of 231 serum samples.

		CMIA		Total
		Positive	Negative	
Proposed method	Positive	108	5	113
	Negative	1	117	118
	Total	109	122	231

**Figure 4:**
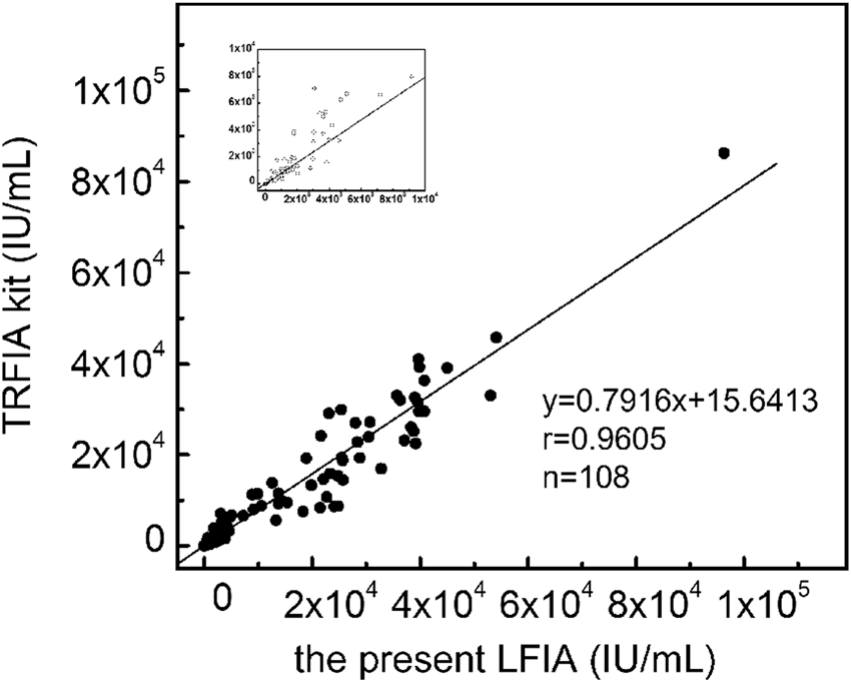
Comparison of anti-HBc levels in 108 anti-HBc positive specimens measured using the developed method and a TRFIA kit.

In addition, for comparison between methods, the 108 anti-HBc positive serum samples were analyzed using both the developed CM-EUs test strip and a TRFIA kit (Darui, Guangzhou, China) (see Supplementary Table S3). As shown in Fig. 4, a good level of agreement between the two methods was observed. The equation for the regression curve was y = 0.7916x + 15.6413, with r values of 0.9605 (n = 108, P < 0.001), where y represents the anti-HBc concentration obtained by the Darui TRFIA kit and x is the concentration obtained using the developed LFIA strip. The results suggest that the anti-HBc Eu (III) chelate microparticle-based LFIA strips are effective for the quantitative determination of anti-HBc in human serum.

## Discussion

Anti-HBc is one of the most classical serological markers during HBV infection and has been widely used in clinical diagnosis or blood screening^4^. More importantly, qAnti-HBc measurements could play an important role in evaluating the therapy and optimising the antiviral therapy of CHB^8–13,28^. However, there are only a few methods to quantitatively analyze anti-HBc level in human serum, and these methods require expensive instrumentation, complexity of the procedures, and long operation times.

It is notable that HBeAg could influence the measurement results when using a double-antigen sandwich format to determine anti-HBc levels in HBeAg positive serum samples. This is because of the instability of HBeAg, which possesses a structure that is like that of the hepatitis B core antigen, and is easily transformed into the hepatitis B core antigen. For this reason, like most commercially available anti-HBc kits that adopt a competitive format, we establish a direct competitive LFIA system for the detection of qAnti-HBc in human serum.

Over the last decade, Eu(III) chelate microparticles-based LFIA has attracted widespread attention^29–31^. In this paper, we first report the development of a simple and rapid point-of-care system, which integrates Eu(III) chelate microparticles with LFIA to quantitative determine anti-HBc in serum. This assay is based on a direct competitive immunoassay performed on lateral flow test strips with an assay time of 15 min. The H_T_/H_C_ ratio was applied to quantify the result of the measurement to offset the effect of sample matrix and the inherent heterogeneity of lateral flow test strips.

The Eu (III) chelate microparticle-based LFIA assay could quantitatively detect anti-HBc levels with a limit of detection of 0.31 IU mL^−1^, and exhibited a wide linear range (0.63–640 IU mL^−1^). Compared with commercially available qAnti-HBc kits, such as the Darui TRFIA kit (dynamic range 0.40–5.00 IU mL^−1^; Darui, Guangzhou, China) and the Wantai ELISA kit (dynamic range 0.08–2.50 IU mL^−1^; Wantai, Beijing, China), the proposed method demonstrates advantages in terms of the maximum measurable concentration of anti-HBc, whereby only a 1/100 to 1/10000 (100-fold increase) dilution is required when the anti-HBc level is >640 IU mL^−1^. This means that in the detection of high anti-HBc concentration samples by using our method, dilution and detection times were less than by using other methods. Moreover, the proposed method had an additional advantages of fast turnaround time (15 min for an entire analysis) compared to other qAnti-HBc methods which need a total assay time of 75 to 125 min.

In summary, we have successfully developed Eu (III) chelate microparticle-based LFIA strips that could provide not only qualitative results for the rapid screening of blood, but also quantitative results which could be used to determine the anti-HBc titer in human serum. The assay is convenient, simple, rapid (only 15 min) and low in cost. The proposed method has the lowest detection limit of the trialed assays at 0.31 IU mL^−1^, and excellent linearity in the range of 0.63 to 620 IU mL^−1^. Test results from 231 samples indicate that the developed method demonstrates high consistency with commercially available diagnostic kits. Considering its advantages and performance, the use of this method for the quantitative detection of anti-HBc could help clinicians more rapidly and expediently undertake screening of blood, monitor the course of HBV infections and predict the response to antiviral treatment.

## Methods

### Reagents and instrumentation

Recombinant Hepatitis B core antigen (HBcAg) (JR05003), anti-HBcAg monoclonal antibody (McAb) (B018) and a qAnti-HBc time resolved fluoroimmunoassay (TRFIA) kit were obtained from Guangzhou Darui Biotechnology Co., Ltd. (Guangzhou, Guangdong, China). An Architect anti-HBc chemiluminescent microparticle immunoassay (CMIA) kit was obtained from Abbott Laboratories (Abbott Park, IL, USA). Rabbit IgG (RIgG) and a goat anti rabbit IgG (anti-RIgG) Fc fragment were purchased from Rockland Immunochemicals Inc. (Limerick, PA, USA). Bovine serum albumin (BSA) was obtained from Roche Diagnostics (Indianapolis, IN, USA). CM-EUs (ø 200 nm) were purchased from Thermo Fisher Scientific Inc. (Waltham, MA, USA). A sample pad was obtained from Jieyi Biotechnology (Shanghai, China). A nitrocellulose (NC) membrane, conjugate pad and absorbent pad were obtained from Millipore (Bedford, MA, USA). Casein-Na, Polyvinyl alcohol (PVA), Polyvinyl pyrrolidone (PVP), 4-morpholineethanesulfonic acid (MES), a centrifugal filter unit with an Ultracel-50 membrane, TritonX-100, Proclin-300, 1-ethyl-3-(3-dimethylaminopropyl) carbodiimide hydrochloride (EDC), and N-hydroxysulfosuccinimide (sulfo-NHS) were purchased from Sigma-Aldrich (St. Louis, MO, USA). Tetronic 1307 was purchased from Hangan Technology Co., Ltd (Hangzhou, Zhejiang, China). Trehalose was purchased from Wako Pure Chemical Industries, Ltd (Chuo-ku, Osaka, Japan). All other chemicals were of analytical reagent grade and ultra-pure water was used throughout the study and was produced using a Milli-Q water purification system (Millipore, Bedford, MA, USA). A probe sonicator, Scientz-IID, was purchased from Ningbo Scientz Biotechnology Co., Ltd (Ningbo, Zhejiang, China). A BioJet Quant XYZ3060 dispenser was purchased from Biodot Ltd (Irvine, CA, USA). An aQcare time-resolved fluorescence (TRF) strip reader was obtained from Medisensor, Inc. (Daegu, Korea).

Buffer solutions used in the study were coating buffer (10 mmol L^−1^ Na_2_HPO_4_·12H_2_O, 0.9% NaCl (wt/vol), 3% trehalose (wt/vol) and 0.1% NaN_3_ (wt/vol), pH 7.4), sample pad treatment buffer (100 mmol L^−1^ Na_2_B_4_O_7_.10H_2_O, 1% PVP (wt/vol), 0.2% casein-Na (wt/vol), 1% TritonX-100 (vol/vol), 1% Tetronic 1307 and 0.1% NaN_3_ (wt/vol)), conjugate pad treatment buffer (50 mmol L^–1^ Na_2_HPO_4_.12H_2_O, 0.5% PVA (wt/vol), 0.5% BSA (wt/vol) and 1% TritonX-100 (vol/vol), pH 7.4), activating buffer (25 mmol L^−1^ MES, pH 6.1), binding buffer (25 mmol L^−1^ phosphate buffer, pH 7.0), blocking buffer (25 mmol L^−1^ phosphate buffer, 2% BSA (wt/vol), pH 7.4), washing buffer (25 mmol L^−1^ Tris-HCl, 0.9% NaCl (wt/vol), 0.2% Tween-20 (vol/vol) and 0.05% Proclin-300 (vol/vol), pH 7.8), labeling antibody dilution buffer (25 mmol/L Tris–HCl, 1% BSA (wt/vol), 5% Trehalose (wt/vol), 20% sucrose (wt/vol) and 0.05% Proclin-300 (vol/vol), pH 9.0) and sample buffer (50 mmol L^−1^ Tris-HCl, 0.1% NaN_3_ (wt/vol), 0.9% NaCl (wt/vol), 0.01% Tween-20 (vol/vol), 7.5% BSA (wt/vol), pH 7.8). All solutions were freshly prepared before use.

### Preparation of CM-EUs coupled with anti-HBcAg McAb or RIgG

The conjugates of CM-EUs and anti-HBcAg McAb were prepared using a method as described in our previous work with only minor modifications^23,24^. In brief, 2 mg of CM-EUs were suspended in 1 mL of activating buffer including sulfo-NHS and EDC with final concentrations of 10 mmol L^−1^ and 1.25 mmol L^−1^, respectively. After reaction with gentle shaking for 0.5 h at room temperature, the mixture was centrifuged at 15000 g for 20 min at 8 °C and the supernatant was removed. Then the activated CM-EUs were washed twice and re-suspended in 1 mL of binding buffer using sonication. Subsequently, 0.05 mg of anti-HBcAg McAb, which was purified and condensed in advance using a centrifugal filter unit with an Ultracel-50 membrane, was mixed with the activated CM-EUs, and then the reaction mixture was vibrated for 2 h at room temperature. The uncoupled antibody was removed via centrifugation at 10000 g for 15 min at 8 °C. After twice washing, 1 mL of blocking buffer was added and shaken for 1 h at room temperature to block unreacted active sites. The supernatant was discarded, the conjugate was washed and sonicated using a microprobe at 5% amplitude for 3 min, and the washing buffer was discarded by centrifugation at 10000 g for 15 min at 8 °C. The washing process was repeated three times. Finally, the conjugates were resuspended in 0.2 mL of labeling antibody dilution buffer yielding a concentration of CM-EUs of 10 g L^−1^ and stored at 4 °C until use. The conjugates of RIgG and CM-EUs were also prepared using a similar method.

### Preparation of CM-EUs-based test strip

The CM-EUs-based test strip consists of five parts, including the sample pad, conjugate pad, NC membrane, absorbent pad and backing plate. Initially, the sample pad and conjugate pad were pretreated with sample pad treatment buffer and conjugate pad treatment buffer, respectively, as described in our previous work^23,24^. The conjugates of CM-EUs with anti-HBcAg McAb and the conjugates of CM-EUs with RIgG were diluted and mixed in antibody dilution buffer with final concentrations of CM-EUs of 0.5 g L^−1^ and 0.025 g L^−1^, respectively. Then the mixed conjugates were dispensed onto the pretreated conjugate pad at a rate of 5 μL cm^−1^ using a BioJet XYZ-3060 Quanti dispenser, and then dried at 37 °C for 2 h. HBcAg (0.5 mg mL^−1^) and anti-RIgG (2 mg mL^−1^), were dissolved in coating buffer, and were dispersed on a NC membrane at a rate of 0.8 μL cm^−1^ as the test line and control line, respectively, being separated by a distance of 4 mm. The NC membrane was then immediately dried at 37 °C for 3 h. In addition, an absorbent pad was used directly. The four parts were laminated on a 300 × 60 mm backing plate in the proper alignment so that there was a continuous flow path for the sample (Fig. 1A). Lastly, the whole plate was cut into 3 mm-wide strips using a strip cutter and placed in strip cassettes. The prepared test strips were stored in a drying oven at room temperature until use.

### Calibration

Anti-HBc levels were determined in IU mL^−1^ and were correlated to the First International Standard (2013) for HBcAg (National Institute for Biological Standards and Control (NIBSC) code: 95/522), being assigned a unitage of 50 IU/ampoule. This certified anti-HBc standard material was used as the gold standard for our laboratory and was serially diluted to calibrate the anti-HBc standard of our polystyrene Eu (III) chelate microparticle-based LFIA. The ratio of the theoretical value to the measured value was between 0.9 and 1.1.

### Samples

Serum samples from a total of 343 patients were kindly provided by Nanfang Hospital (Guangzhou, Guangdong, China) and Fuzhou Infectious Hospital (Fuzhou, Fujian, China). Samples included those from 112 healthy people, 134 individuals undergoing their annual health check and 97 CHB patients. All samples were stored at −20 °C until use. The study was approved by the ethics committees of Nanfang Hospital and the ethics committees of Fuzhou Infectious Hospital. The methods for sample collection and experiments were carried out in accordance with the principles stated in the Declaration of Helsinki. Written informed consent was obtained from each patient.

### Fluorescence lateral flow assay procedure

First, 60 μL of the standards or samples was added to 60 μL of the sample buffer and 60 μL of the mixture was loaded onto the sample pad well and the liquid migrated towards the absorption pad by capillary action. After a 15-min reaction, the cassette containing the test strip was inserted and scanned using the aQcare TRF reader. The H_T_ and H_C_ were measured, and the ratio of H_T_/H_C_ was recorded. A schematic illustration of the polystyrene Eu (III) chelate microparticle-based LFIA procedure is shown in Fig. 1.

### Statistical analysis

The anti-HBc standard curve was obtained by plotting the logit-log against the logarithm of the corresponding anti-HBc concentration (X). The H_T_/H_C_ ratios between the zero anti-HBc standard and the other anti-Hbc standards were defined as B_o_ and B_x_, respectively. A logit-log plot was obtained from the computation formula: logit (Y) = ln [(B_x_/B_0_)/(1 − B_x_/B_0_)]. The curves and logistic equations were obtained by fitting the line of best fit using Origin Pro7.5 (GE, Piscataway, NJ, USA), which was logit (Y) = A + B × log (X). Signal linearity and correlations were checked using Pearson’s linear regression equation. McNemar’s test and Pearson’s correlation were performed to show the consistency and correlation between two methods using SPSS 13.0 (Chicago, IL, USA). P < 0.05 was considered statistically significant. Sample means and standard deviations (SD) were determined using Microsoft Excel (version 2.12; Analyse-it Ltd, Leeds, UK).

### Data Availability

All data generated or analysed during this study are included in this published article.

## Electronic supplementary material

All the data of each serum samples in the section of Anti-HBc measurements in serum samples
