# Supplementary material for: Europium (III) chelate microparticle-based lateral flow immunoassay strips for rapid and quantitative detection of antibody to hepatitis B core antigen
Source: Sci Rep. 2017 Oct 26;7:14093. doi: 10.1038/s41598-017-14427-4 (PMC5658374; doi:10.1038/s41598-017-14427-4)
Supplement: Supplementary file 1 — All the data of each serum samples in the section of Anti-HBc measurements in serum samples [file 41598_2017_14427_MOESM1_ESM.pdf]

Europium (III) chelate microparticle-based lateral flow immunoassay strips for rapid and quantitative detection of antibody to hepatitis B core antigen

Rong-Liang Liang, Qiao-Ting Deng, Zhen-Hua Chen, Xu-Ping Xu, Jian-Wei Zhou, Jun-Yu Liang, Zhi-Ning Dong1, Tian-Cai Liu1, and Ying-Song Wu

Supplementary Table S1

Sample	Proposed method (IU mL^{-1})	Sample	Proposed method (IU mL^{-1})	Sample	Proposed method (IU mL^{-1})
1	＜0.31	39	＜0.31	77	0.63
2	＜0.31	40	＜0.31	78	＜0.31
3	＜0.31	41	＜0.31	79	＜0.31
4	＜0.31	42	＜0.31	80	＜0.31
5	＜0.31	43	＜0.31	81	＜0.31
6	0.35	44	＜0.31	82	0.41
7	＜0.31	45	＜0.31	83	＜0.31
8	＜0.31	46	＜0.31	84	＜0.31
9	＜0.31	47	＜0.31	85	＜0.31
10	＜0.31	48	0.46	86	＜0.31
11	＜0.31	49	＜0.31	87	＜0.31
12	＜0.31	50	＜0.31	88	＜0.31
13	0.93	51	＜0.31	89	＜0.31
14	＜0.31	52	＜0.31	90	1.66
15	＜0.31	53	0.98	91	＜0.31
16	＜0.31	54	＜0.31	92	＜0.31
17	＜0.31	55	＜0.31	93	＜0.31
18	＜0.31	56	＜0.31	94	＜0.31
19	＜0.31	57	＜0.31	95	＜0.31
20	1.34	58	1	96	＜0.31
21	＜0.31	59	＜0.31	97	＜0.31
22	＜0.31	60	＜0.31	98	0.79
23	＜0.31	61	＜0.31	99	＜0.31
24	＜0.31	62	＜0.31	100	＜0.31
25	＜0.31	63	1.16	101	＜0.31
26	＜0.31	64	0.84	102	＜0.31

27	0.57	65	＜0.31	103	1.83
28	＜0.31	66	＜0.31	104	＜0.31
29	＜0.31	67	＜0.31	105	＜0.31
30	＜0.31	68	＜0.31	106	＜0.31
31	1.25	69	＜0.31	107	＜0.31
32	＜0.31	70	＜0.31	108	＜0.31
33	＜0.31	71	0.38	109	0.42
34	＜0.31	72	＜0.31	110	＜0.31
35	＜0.31	73	＜0.31	111	＜0.31
36	0.42	74	＜0.31	112	＜0.31
37	＜0.31	75	＜0.31		
38	0.78	76	＜0.31		

Supplementary Table S2

Sample	CMIA	Proposed method (IU mL^{-1})	Sample	CMIA	Proposed method (IU mL^{-1})
113	Negetive	＜0.31	229	Negetive	＜0.31
114	Negetive	＜0.31	230	Negetive	＜0.31
115	Negetive	＜0.31	231	Negetive	2.14
116	Negetive	＜0.31	232	Negetive	＜0.31
117	Negetive	＜0.31	233	Negetive	＜0.31
118	Negetive	＜0.31	234	Negetive	＜0.31
119	Negetive	＜0.31	235	Positive	18763.05
120	Positive	11234.02	236	Negetive	＜0.31
121	Negetive	＜0.31	237	Negetive	＜0.31
122	Negetive	1.26	238	Negetive	＜0.31
123	Negetive	＜0.31	239	Negetive	＜0.31
124	Negetive	＜0.31	240	Negetive	＜0.31
125	Negetive	＜0.31	241	Negetive	＜0.31
126	Negetive	＜0.31	242	Negetive	＜0.31
127	Negetive	＜0.31	243	Negetive	＜0.31
128	Positive	973.02	244	Negetive	＜0.31
129	Negetive	＜0.31	245	Negetive	＜0.31
130	Negetive	＜0.31	246	Negetive	＜0.31
131	Negetive	＜0.31	247	Positive	3859.47
132	Positive	33000.01	248	Positive	1081.70
133	Positive	5.70	249	Positive	6687.49

134	Negetive	＜0.31	250	Positive	7101.71
135	Negetive	＜0.31	251	Positive	6245.09
136	Negetive	＜0.31	252	Positive	8365.35
137	Negetive	3.11	253	Positive	325.44
138	Negetive	＜0.31	254	Positive	5351.60
139	Negetive	＜0.31	255	Positive	9286.54
140	Negetive	＜0.31	256	Positive	6624.88
141	Negetive	＜0.31	257	Positive	1846.23
142	Positive	15.03	258	Positive	905.18
143	Negetive	＜0.31	259	Positive	5625.88
144	Negetive	＜0.31	260	Positive	14652.31
145	Negetive	＜0.31	261	Positive	718.32
146	Negetive	＜0.31	262	Positive	241.41
147	Negetive	＜0.31	263	Positive	5206.67
148	Negetive	＜0.31	264	Positive	1069.17
149	Negetive	＜0.31	265	Positive	888.03
150	Negetive	＜0.31	266	Positive	8612.87
151	Negetive	＜0.31	267	Positive	29582.57
152	Negetive	＜0.31	268	Positive	19206.52
153	Negetive	＜0.31	269	Positive	7985.86
154	Negetive	＜0.31	270	Positive	10016.34
155	Negetive	＜0.31	271	Positive	888.03
156	Negetive	＜0.31	272	Positive	1563.22
157	Negetive	＜0.31	273	Positive	25.84
158	Negetive	＜0.31	274	Positive	365.64
159	Negetive	2.61	275	Positive	5329.26
160	Positive	39000.10	276	Positive	1778.07
161	Negetive	＜0.31	277	Positive	1161.87
162	Negetive	＜0.31	278	Positive	14487.00
163	Negetive	＜0.31	279	Positive	8758.00
164	Negetive	＜0.31	280	Positive	19438.70
165	Negetive	＜0.31	281	Positive	19318.95
166	Negetive	＜0.31	282	Positive	771.41
167	Negetive	＜0.31	283	Positive	1093.98
168	Negetive	＜0.31	284	Positive	11524.94
169	Negetive	＜0.31	285	Positive	873.56
170	Negetive	＜0.31	286	Positive	7502.26
171	Negetive	＜0.31	287	Positive	13242.48
172	Negetive	＜0.31	288	Positive	15396.81
173	Negetive	＜0.31	289	Positive	23854.97

174	Negetive	＜0.31	290	Positive	31.52
175	Negetive	＜0.31	291	Positive	572.00
176	Positive	102.04	292	Positive	16931.57
177	Negetive	＜0.31	293	Positive	961.51
178	Negetive	＜0.31	294	Positive	25147.17
179	Negetive	＜0.31	295	Positive	1992.99
180	Negetive	＜0.31	296	Positive	9532.43
181	Negetive	＜0.31	297	Positive	409.39
182	Negetive	＜0.31	298	Positive	3272.85
183	Negetive	＜0.31	299	Positive	3757.80
184	Negetive	＜0.31	300	Positive	19.61
185	Negetive	1.82	301	Positive	27166.60
186	Negetive	＜0.31	302	Positive	3168.19
187	Negetive	＜0.31	303	Positive	3849.30
188	Positive	47.02	304	Positive	29104.89
189	Negetive	＜0.31	305	Positive	36312.62
190	Negetive	＜0.31	306	Positive	3723.47
191	Negetive	＜0.31	307	Positive	26066.15
192	Negetive	＜0.31	308	Positive	233.62
193	Negetive	＜0.31	309	Positive	40940.60
194	Negetive	＜0.31	310	Positive	3226.73
195	Negetive	＜0.31	311	Positive	4374.43
196	Negetive	＜0.31	312	Positive	24106.96
197	Negetive	＜0.31	313	Positive	22474.89
198	Positive	210.01	314	Positive	1648.41
199	Negetive	＜0.31	315	Positive	10719.82
200	Negetive	＜0.31	316	Positive	4.50
201	Negetive	＜0.31	317	Positive	248.41
202	Negetive	＜0.31	318	Positive	1041.33
203	Negetive	＜0.31	319	Positive	22778.96
204	Negetive	＜0.31	320	Positive	1871.82
205	Negetive	＜0.31	321	Positive	5007.32
206	Negetive	＜0.31	322	Positive	23132.01
207	Negetive	＜0.31	323	Positive	27018.71
208	Positive	0.92	324	Positive	15826.75
209	Negetive	＜0.31	325	Positive	31446.58
210	Negetive	＜0.31	326	Positive	33047.54
211	Negetive	＜0.31	327	Positive	967.46
212	Negetive	＜0.31	328	Positive	236.37
213	Negetive	＜0.31	329	Positive	86245.69

214	Negetive	＜0.31	330	Positive	1743.80
215	Negetive	＜0.31	331	Positive	8728.72
216	Positive	1302.03	332	Positive	29875.46
217	Negetive	＜0.31	333	Positive	29541.85
218	Negetive	＜0.31	334	Positive	514.96
219	Negetive	＜0.31	335	Positive	45754.15
220	Negetive	＜0.31	336	Positive	13797.61
221	Negetive	＜0.31	337	Positive	39197.91
222	Negetive	＜0.31	338	Positive	182.09
223	Negetive	＜0.31	339	Positive	1176.62
224	Negetive	＜0.31	340	Positive	743.37
225	Negetive	＜0.31	341	Positive	32051.95
226	Negetive	＜0.31	342	Positive	32574.09
227	Negetive	＜0.31	343	Positive	11434.27
228	Negetive	＜0.31			

Supplementary Table S3

Sample	TRFIA (IU mL^{-1})	Proposed method (IU mL^{-1})	Sample	TRFIA (IU mL^{-1})	Proposed method (IU mL^{-1})
120	8937	11234.02	290	110	31.52
128	1399	973.02	291	1040	572.00
132	53000	33000.01	292	32800	16931.57
133	3.4	5.7	293	1090	961.51
142	12	15.03	294	38800	25147.17
160	45000	39000.1	295	1650	1992.99
176	87	102.01	296	15400	9532.43
188	56	47.02	297	420	409.39
198	157	210.01	298	4000	3272.85
216	2005	1302.03	299	1800	3757.80
235	25643	18763.05	300	36	19.61
247	1820	3859.47	301	30700	27166.60
248	1020	1081.70	302	3020	3168.19
249	5100	6687.49	303	3050	3849.30
250	3060	7101.71	304	23100	29104.89

251	4740	6245.09	305	40800	36312.62
252	21500	8365.35	306	3640	3723.47
253	940	325.44	307	38300	26066.15
254	3300	5351.60	308	430	233.62
255	13800	9286.54	309	39700	40940.60
256	7200	6624.88	310	4620	3226.73
257	2980	1846.23	311	4190	4374.43
258	1150	905.18	312	21600	24106.96
259	13300	5625.88	313	39200	22474.89
260	22100	14652.31	314	1520	1648.41
261	830	718.32	315	22700	10719.82
262	640	241.41	316	1.5	4.50
263	3620	5206.67	317	430	248.41
264	1440	1069.17	318	1710	1041.33
265	1390	888.03	319	28400	22778.96
266	24100	8612.87	320	1830	1871.82
267	40800	29582.57	321	3640	5007.32
268	18900	19206.52	322	37100	23132.01
269	9200	7985.86	323	28000	27018.71
270	14300	10016.34	324	23300	15826.75
271	540	888.03	325	39600	31446.58
272	3840	1563.22	326	35700	33047.54
273	14.4	25.84	327	440	967.46
274	1070	365.64	328	390	236.37
275	3780	5329.26	329	96300	86245.69
276	1200	1778.07	330	720	1743.80
277	1380	1161.87	331	24800	8728.72
278	25700	14487.00	332	25400	29875.46
279	10600	8758.00	333	39800	29541.85
280	25500	19438.70	334	840	514.96
281	28900	19318.95	335	54100	45754.15
282	950	771.41	336	12600	13797.61
283	1130	1093.98	337	39900	39197.91
284	13800	11524.94	338	370	182.09
285	1210	873.56	339	2830	1176.62
286	18300	7502.26	340	2020	743.37
287	19900	13242.48	341	36300	32051.95
288	24800	15396.81	342	39000	32574.09
289	30500	23854.97	343	9900	11434.27
